# Deciphering the Prokaryotic Community and Metabolisms in South African Deep-Mine Biofilms through Antibody Microarrays and Graph Theory

**DOI:** 10.1371/journal.pone.0114180

**Published:** 2014-12-22

**Authors:** Yolanda Blanco, Luis A. Rivas, Antonio García-Moyano, Jacobo Aguirre, Patricia Cruz-Gil, Arantxa Palacín, Esta van Heerden, Víctor Parro

**Affiliations:** 1 Department of Molecular Evolution, Centro de Astrobiología (INTA-CSIC), Carretera de Ajalvir, km 4, Torrejón de Ardoz, 28850, Madrid, Spain; 2 TIA/UFS Metagenomics Platform, Department of Biotechnology, University of the Free State, P. O. Box 339, Bloemfontein, 9300, South Africa; 3 Centro de Biotecnología y Genómica de Plantas, Campus de Montegancedo, Autopista M40, km 38, 28223, Pozuelo de Alarcón, Madrid, Spain; J. Craig Venter Institute, United States of America

## Abstract

In the South African deep mines, a variety of biofilms growing in mine corridor walls as water seeps from intersections or from fractures represents excellent proxies for deep-subsurface environments. However, they may be greatly affected by the oxygen inputs through the galleries of mining activities. As a consequence, the interaction between the anaerobic water coming out from the walls with the oxygen inputs creates new conditions that support rich microbial communities. The inherent difficulties for sampling these delicate habitats, together with transport and storage conditions may alter the community features and composition. Therefore, the development of in situ monitoring methods would be desirable for quick evaluation of the microbial community. In this work, we report the usefulness of an antibody-microarray (EMChip66) immunoassay for a quick check of the microbial diversity of biofilms located at 1.3 km below surface within the Beatrix deep gold mine (South Africa). In addition, a deconvolution method, previously described and used for environmental monitoring, based on graph theory and applied on antibody cross-reactivity was used to interpret the immunoassay results. The results were corroborated and further expanded by 16S rRNA gene sequencing analysis. Both culture-independent techniques coincided in detecting features related to aerobic sulfur-oxidizers, aerobic chemoorganotrophic *Alphaproteobacteria* and metanotrophic *Gammaproteobacteria*. 16S rRNA gene sequencing detected phylotypes related to nitrate-reducers and anaerobic sulfur-oxidizers, whereas the EMChip66 detected immunological features from methanogens and sulfate-reducers. The results reveal a diverse microbial community with syntrophic metabolisms both anaerobic (fermentation, methanogenesis, sulphate and nitrate reduction) and aerobic (methanotrophy, sulphur oxidation). The presence of oxygen-scavenging microbes might indicate that the system is modified by the artificial oxygen inputs from the mine galleries.

## Introduction

The deep subsurface has attracted much interest to unravel the microbial strategies to cope with an environment characterized by limited nutrient availability, high temperature and pressure. Microbial inhabitants in deep subsurface represent a large proportion of the biomass on Earth [1]. The knowledge of this largely-unexplored microbial diversity may provide relevant findings for microbial ecology as well as for potential biotechnological applications. Ultra-deep mines provide an easy access to ultra-deep microbiota [2]. Several studies by culture-dependent and independent methods have been conducted on different microbial habitats in gold mines in Japan [3], [4] and North America [5]. The ultra-deep habitats located in the South African mines (Witwatersrand Basin) have also been the subject of several geochemical and microbiological studies. Different and often massive microbial growths frequently occur in corridors and passages in deep mines when the water drips from open exploratory boreholes or the intersections with water-bearing fractures during mining operations. Their microbial populations have been found to reflect the geochemistry of the water. Additionally, high concentrations of contaminating organisms find difficult to out-compete the indigenous microorganisms once the original geochemical conditions have been restored [6], [7] and novel microorganisms have been found to inhabit these biofilms [7]. Whereas the biogeochemical characteristics and prokaryotic diversity in fracture and service waters from South African mines have been extensively studied [2], [6], [8], [9], [10], [11], [12] the biofilms located on the mine walls have remained relatively under-studied. Phospholipid fatty acid (PLFA) analysis was performed in biofilms from eight different mines [11], and molecular phylogeny by 16S rRNA gene sequencing was determined in a biofilm from a borehole outlet [8].

Sampling in extreme environments is often complicated and the amount and the number of samples are not always as the researchers need. In addition, sample collection, transportation and storage may introduce alteration to the original microbial composition and activities. Also, each molecular ecology technique has its own drawbacks, mainly due to the multiples steps to obtain the final result. For example, the results from DNA sequencing (massive or not) are affected by the initial sample amount, its preservation during transport, the lysis efficiency, the DNA yield, or the PCR amplification and library biases (for cloning or massive sequencing). We have reported recently how important is a multi-technique approach to study the microbial diversity and metabolic processes in extreme low-cell density subsurface habitats [13]. In these scenarios, antibody microarrays can contribute to capture new features that escape to other techniques, such as the detection of biomarkers from death cells, spores or other resistant (difficult to lyse) cells, certain polymeric compounds in a microbial biofilm, or a particular protein or toxins. Microarray immunoassays are robust and easy to perform even in the field, permitting a fast evaluation of the main characteristics of the microbial community and can help to take decision for further sampling [14], [15], [16], [17]. However, the number of microbes it can detect is limited, even more, the identification is not always easy due to the cross-reactions between antibodies. New improvements in the technology [18], [19], [20] and data analysis based on graph theory and associated deconvolution method [21] allow to scale up the number of antibodies and targets assayed simultaneously. We modeled a 66-antibody microarray for sandwich immunoassays and its antibody cross-reactivity events as a directed and weighted network named as *antibody graph* [21]. In an antibody graph, nodes represent antibodies and links represent cross-reactivity for the antigenic sample between the tracer and the capturing antibody. In the same work, we reported a deconvolution method, based on the concept of antibody graph, which gave qualitative estimation of the composition of environmental microbial communities by distinguishing cross-reactivity events from the cognate antigen-antibody reactions. The deconvolution analysis can be applied to both *closed systems*, that is, those for which all the analytes present in the samples were used as cognate immunogens for producing, at least, one antibody used in the immunoassay, and *open systems*, those that contain analytes that were not used as immunogens. Environmental samples like those of the South African mines studied in the present work are open systems (tens of cm to m scale) and the deconvolution method yields not only valuable information about the existence or absence of the cognate antigens of the capturing antibodies, but also about the presence of closely related antigens whose cognate antibodies are not present in the microarray.

Herein, we report the community structure of longitudinal transects of two massive biofilms from a deep gold mine in South Africa. The study was performed by culture-independent analysis using antibody microarray immunoassays, an antibody graph and the deconvolution method associated to it, as well as confirmation of the results with by DNA sequencing. We show how antibody microarray and DNA sequencing supplement each other to gain new information about the biofilms. We concluded that, although there are important anaerobic processes such as methanogenesis, sulfate and nitrate reduction in these biofilms, oxygen-consuming metabolisms (methanotrophy and sulphur oxidation) that might be in syntrophy with the former are also relevant contributors to the microbial community.

## Results

### Geochemical environment and biochemical characterization of deep-subsurface biofilms

Two massive (>50 cm long) biofilms (BF1 and BF2) visually different in colour and texture were collected. They were located in the same corridor within Beatrix Au mine, shaft 3, level 26 at 1.3 km below surface (for details see Experimental Procedures). BF1 showed a black coloration whereas the larger BF2 showed a white colour on the top part and a pink colour towards the bottom. Fissure-associated water flows from two flanking boreholes located within the same corridor. These two boreholes have been already reported [22]. The geochemical characteristics of the fracture water from those boreholes (BH1 and BH2), that could eventually irrigate the biofilms, were determined (Table 1). Temperature values were similar in both water samples and were in the range for mesophilic microorganism growing and both pH values were mildly alkaline. The low oxidation-reduction potential (Eh) values were indicative of a reduced state of both water samples. Chloride concentration values were very different and indicated that BH2 fracture water was more saline than that of BH1. The total organic carbon (TOC) was low, characteristic of oligotrophic water. Similar TOC and DOC (dissolved organic carbon) values in BH2 sample indicated that nearly all carbon content was solubilized. A concentration of 0.01 mM formaldehyde, as a component of DOC, was detected in BH1.

**Table 1:** Summary of the geochemical data from the fracture-associated water collected from BH1 and BH2 boreholes.

Water field measurements	BH1	BH2
T (°C)	30	37.2
pH	7.7	7.9
Eh (mV)	−10	−53.7
TOC (mM)	NM	0.15
DOC (mM)	NM	0.14
TDS (ppm)	4269	3579
Conductivity (mS cm^−1^)	8.54	7.53
Chloride (mM)	5.6	67.5
Nitrate (µM)	<8.0	<7.2
Sulfide (mM)	NM	>10
Sulfate (mM)	<2.0	0.17
Iron (µM)	1.8	<0.23
Formaldehyde (mM)	0.01	NM

Eh: oxidation-reduction potential; TDS: total dissolved solids; TOC: total organic carbon; DOC: dissolved organic carbon. NM: not measured.

Due to the difference in size, three (BF1a, BF1b and BF1c) and six (BF2a, BF2b, BF2c, BF2d, BF2e and BF2f) vertical transect samples were collected by sectioning BF1 and BF2 from the bottom to the top part. Extracts from every vertical transect sample were obtained as described in Experimental Procedures and their total protein and sugar content were measured (S1 Table). Analyses showed an increasing concentration of both components with the water flow, from the top to the bottom. Also, the metal concentration of one lyophilized transect sample from BF1 (BF1c) and two from BF2 (BF2c and BF2e) were analysed by ICP-MS. As shown in Table 2, BF1c sample accumulated the highest amount of Mn, Zn, Cd and Pb in comparison to their respective concentrations in BF2c and BF2e, whereas the Cr, Fe, Cu, Ni and As concentration in BF1c were much lower than those in BF2c and BF2e.

**Table 2:** Metal content (ppm) of the transect biofilm samples analyzed by ICP-MS.

	Cr	Mn	Fe	Co	Ni	Cu	Zn	As	Cd	Pb
BF2c	118.9	1126.4	47787.3	27.5	100.2	132.7	244.7	24.0	0.14	140.0
BF2e	3871.5	4517.6	136189.4	136.2	747.4	142.5	341.2	48.4	0.08	217.9
BF1c	17.7	117755.3	4522.3	14.0	22.4	17.5	3620.1	6.5	5.92	1015.6

### Biofilm characterization by multiplex antibody microarray (EMChip66)

**Figure 1:**
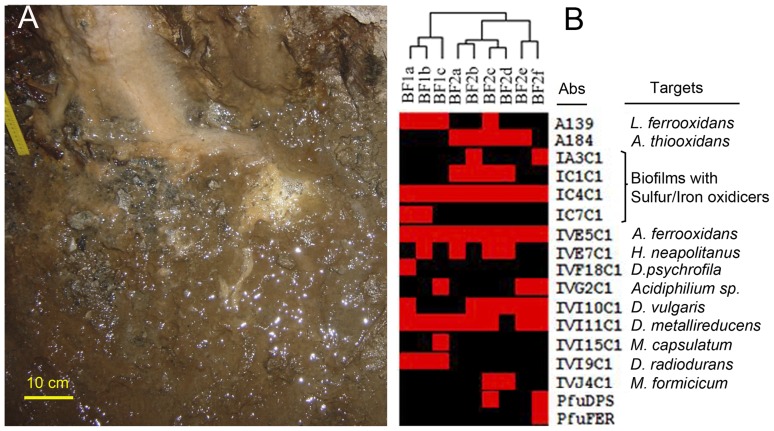
Studying deep South African mine biofilms by antibody microarrays. (A) a photograph of the biofilms on the corridor walls. Different colors might indicate different microbial communities and metabolisms. (B) Heat map representation of the immunoassay results from the longitudinal transects of BF1 and BF2 biofilms (BF1a, BF1b and BF1c, and BF2a, BF2b, BF2c, BF2d, BF2e and BF2f, respectively). The experimental filtered fluorescence intensity data are plotted in red for positive immunodetection and black for negative. The two major clusters of samples obtained by unsupervised hierarchically clustering grouped within the biofilm from which they were sampled.

The microbial communities and the metabolism operating in BF1 and BF2 biofilms were determined by using a multiplex antibody microarray (EMChip66; [21]) in a sandwich immunoassay format. The immunogens for the 66 antibodies corresponded to extracts from environmental samples, microbial cell cultures, and some purified proteins (see Experimental Procedures; S2 Table). To estimate the microbial population heterogeneity of the biofilms, we collected vertical transect samples. Different amounts of sonicated crude extracts, regarding their protein content, were immunoassayed with EMChip66. The fluorescent intensity values from those experiments that render the maximum number of positive antibody spot signals in a non-saturating fluorescence signal conditions were transformed into a matrix and visualized as a heat map (Fig. 1). The immunoassay results showed the heterogeneity expected between the two biofilms (they had different color and appearances detected *de visu*) and also some heterogeneity among the transect samples from the same biofilm. However, the unsupervised hierarchically clustering assembled the transect samples into two major clusters that coincide with the biofilms from where were collected (Fig. 1). All biofilm extracts rendered different positive immunoreactions with antibodies to cell cultures and environmental samples related to sulfur-oxidizers (see S2 Table). From those, only the IC4C1 and the IVE5C1 antibodies gave positive reactions in all transect samples from both biofilms. These antibodies were raised against a biofilm from a sulfur-rich environment sample and against a sulfur-oxidizing bacterium, respectively (S2 Table). Moreover, the IVE7C1 antibody to *Halothiobacillus neapolitanus* gave a positive reaction with one out of three transect samples from BF1 (BF1b) and 3 out of 6 samples from BF2 (BF2a, BF2c and BF2d). The IA3C1 and the IC1C1 antibodies, to other sulfur-oxidizer microbial communities, as well as, the A184 antibody, to a cell culture, gave positive reaction with different transect samples only from BF2. The IC7C1, to a sulfur-oxidizer microbial community, rendered a positive reaction only with BF1a and BF1b transect samples. The IVG2C1 antibody to an aerobic heterotrophic alphaproteobacterium, gave positive reactions with BF1c, Bf2e and BF2f transect samples. Six out of nine transect samples from both biofilms (BF1a and BF2b, BF2c, BF2d, BF2e and BF2f) rendered a positive reaction with the IVI10C1 antibody, to the sulfate-reducer deltaproteobacterium, *Desulfovibrio vulgaris* subsp. vulgaris; and BF1a also with IVF18C1 to the sulfate-reducer deltaproteobacterium, *Desulfotalea psychrophila*. The IVI11C1 positive signal was detected in all the transect samples except that from BF2d. This antibody was raised to *Geobacter sulfurreducens*, a metal-reducer deltaproteobacterium. BF1c was the only biofilm transect that rendered a positive reaction in the IVI15C1 spot, corresponding to an antibody to a methanotrophic bacterium (*Methylomicrobium capsulatum*). Only the transect samples from BF1 showed positive reaction with the IVI9C1 antibody, to *Deinococcus radiodurans*, a member of the group *Deinococcus-Thermus*. Two samples from BF2, Bf2c and BF2d, gave a positive reaction with the IVJ4C1 antibody, to a methanogenic archaea (*Methanobacterium formicicum*). Interestingly, BF2e rendered positive reactions in antibodies to a ferritin protein (PfuFer) and to a DPS-like protein (PfuDPS) whereas BF2c only gave a positive reaction in the latter antibody spot. Both proteins purified from *Pyrococcus furiosus* and used as immunogens, are involved in the homeostasis of iron in prokaryotes (Andrews, 2010). No positive signals were detected either in BF1 or in BF2 from antibody spots related to psychrophilic, halophilic and hyperthermophilic environments, in agreement with the environmental characteristics of the samples.

### Graph-based deconvolution analysis of microarray immunoassay data

**Figure 2:**
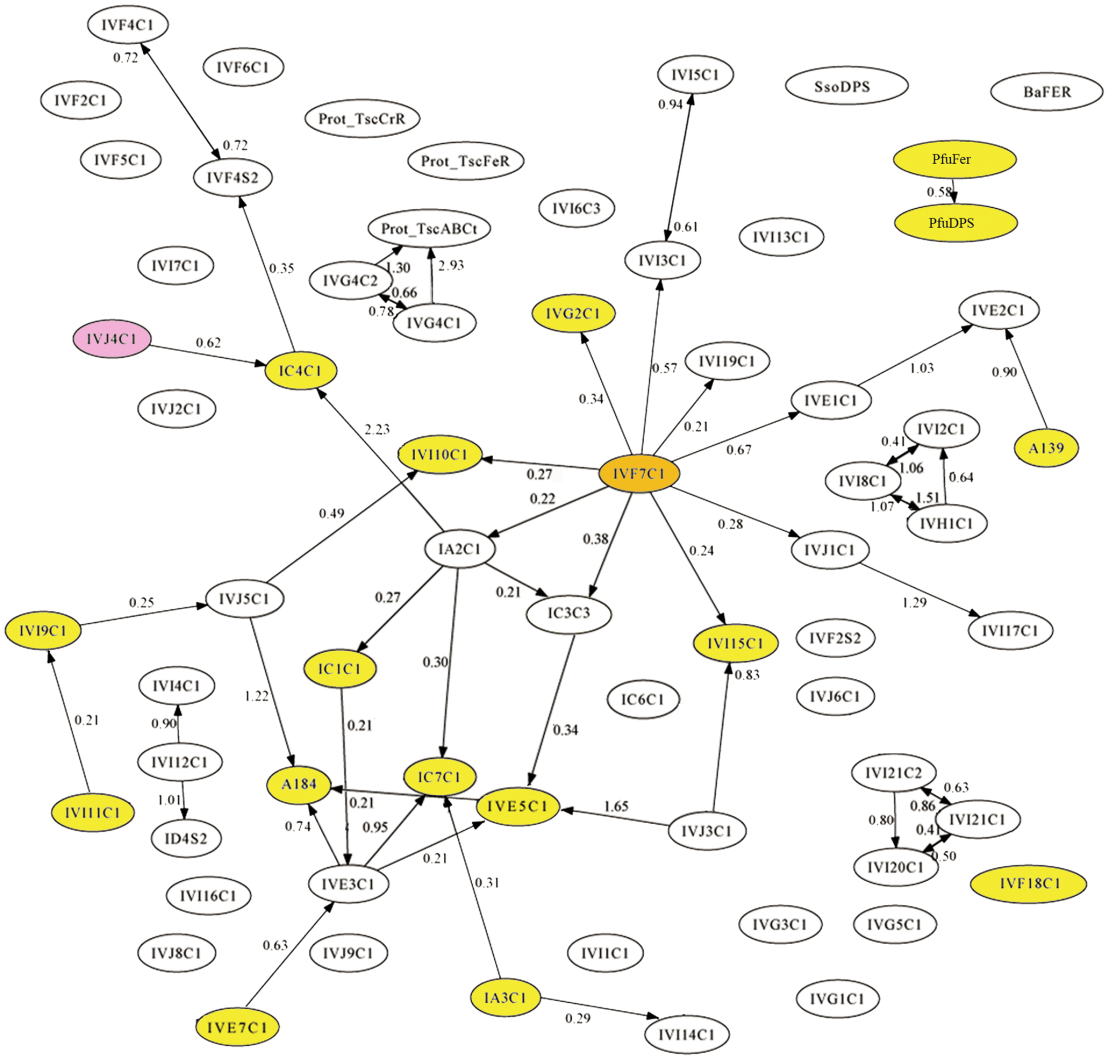
Mapping the positive immunodetections on the antibody graph *G* with 66 nodes and 125 links associated to our EMChip66 antibody microarray. Each node represents one antibody, and the links (arrows) represent cross-reactivity of weight *G_ij_* [21]. Up to 18 colored nodes represent those antibody spots that rendered positive fluorescence in at least one biofilm extract. Self-loops are not shown for clarity. Prot_PfuFer and Prot_PfuDPS =  PfuFer and PfuDPS antibodies respectively.

**Figure 3:**
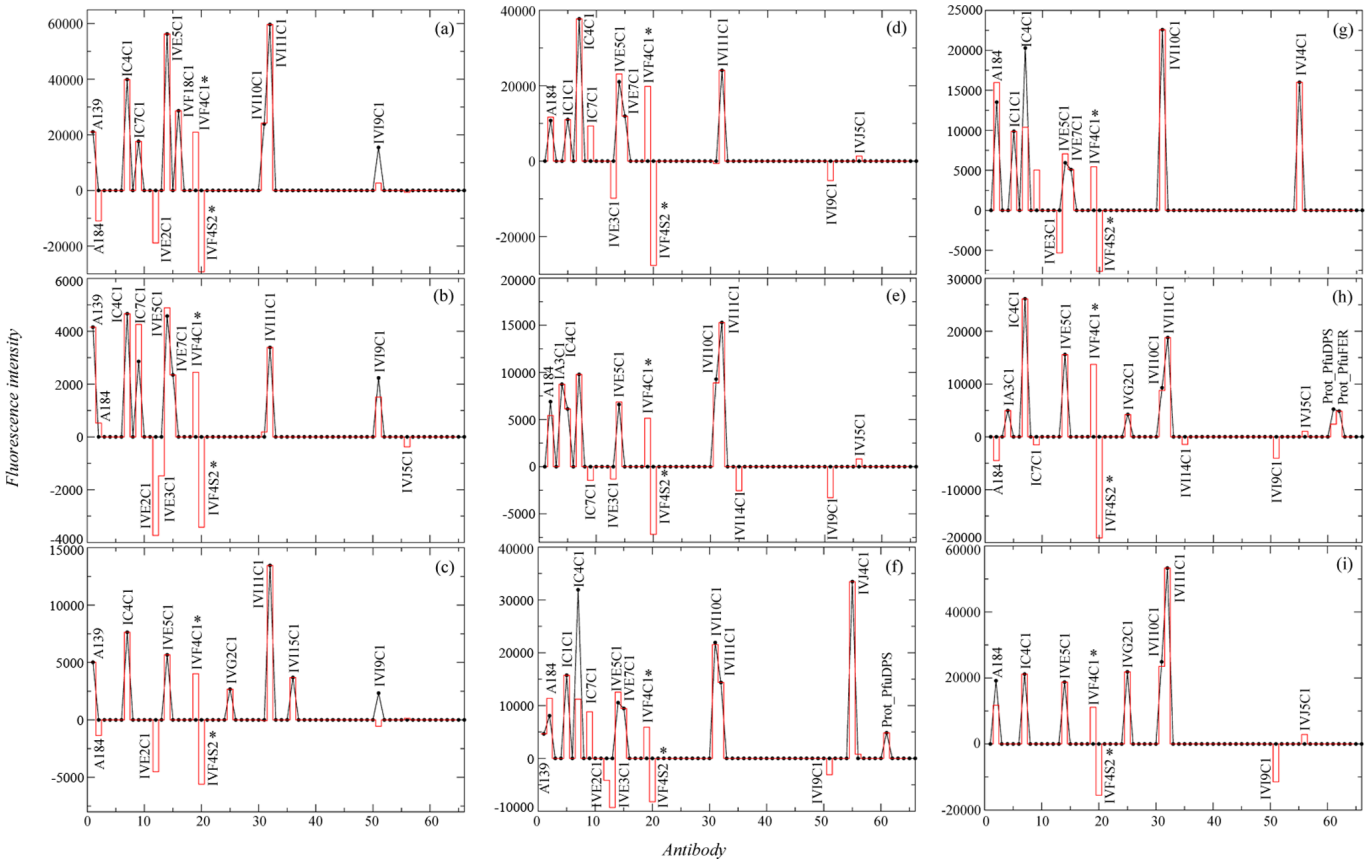
Deconvolution applied to sandwich microarray immunoassays from BF1a (a), BF1b (b), BF1c (c), BF2a (d), BF2b (e), BF2c (f), BF2d (g), BF2e (h) and BF2f (i) transect biofilm extracts. Black lines represent the experimental fluorescence intensities and red lines represent the deconvoluted signals. Antibodies are numbered according to the list shown in S2 Table. Antibodies marked with asterisks represent spurious results (for details see ref. [21]).

**Figure 4:**
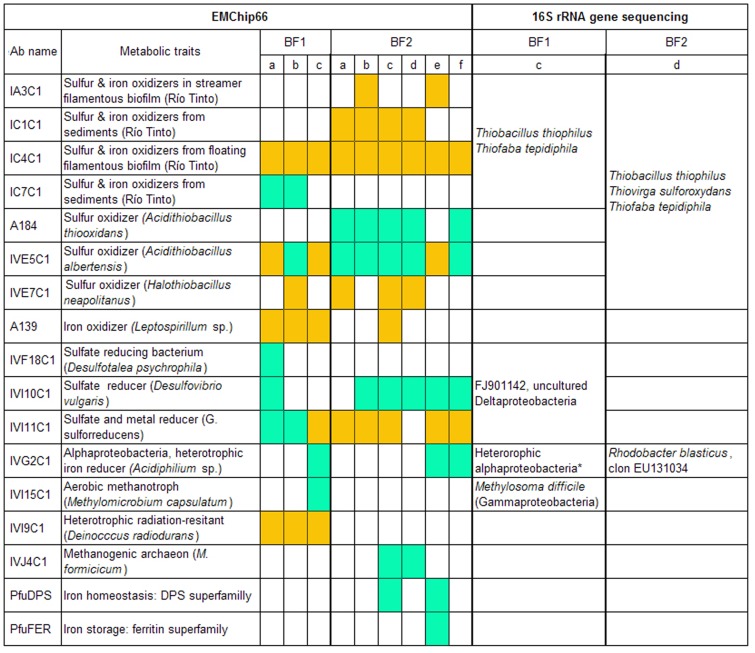
Comparison of EMChip66-deconvoltion results and 16S rRNA gene sequencing. Only antibodies showing positive immunological antigen-antibody interactions in at least one of the transect biofilm samples are listed. *Type I antibodies*: their cognate antigen is not present in the sample (white squares). *Type II antibodies*: their cognate antigen is not present in the sample, but a closely related antigen is present (orange squares). *Type III antibodies*: either their cognate antigen or a closely related one is present in the sample (green squares). Some of the 16S rRNA genes sequences obtained from BF1c and BF2d confirm the results obtained by deconvolution method. * *Parvularcula* sp., *Hyphomonas* sp., *Stappia* sp., *Porphyrobacter* sp.

To obtain additional information about the antigens contained in the biofilm samples, a deconvolution analysis, based on the antibody graph previously defined for EMChip 66 was applied to the experimental filtered microarray results (Fig. 2; [21]). Deconvoluted results estimate the fraction of the fluorescence intensity that is only due to the binding of the antibody to its cognate antigen. Therefore, deconvoluted result below its corresponding experimental fluorescence intensity indicates that other cross-reacting antibody specific bindings contribute to the experimental value. Fig. 3 shows the immunograms obtained by plotting the experimental fluorescence intensity ***F*** (black lines) and their corresponding deconvoluted data F^*^ (red lines) of all the antibody spots. By analyzing the experimental and the deconvoluted values together with the cross-reactivity network enclosed in the antibody graph, it can be inferred, in a way described in Experimental Procedures, whether an experimental fluorescent signal came indeed from its cognate antigen (microorganism) or from a closely related one. Because we are dealing with environmental samples containing multiple analytes that were not used as immunogens for producing the antibodies included in the microarray (what we have called an *open system*), the deconvolution method gives rise to three different possibilities for each antibody printed in the microarray: (type I) its cognate antigen is not present in the sample, (type II) its cognate antigen is not present in the sample, but a related antigen it is, and (type III) either its cognate antigen or a closely related antigen is present in the sample. Fig. 4 compiles all the information obtained from the deconvolution method for each positive antigen-antibody binding in at least one biofilm transect. The cases of the IVI11C1 antibody (raised against *Geobacter metallireducens*) and the IVI15C1 (raised against *Methylomicrobium capsulatum*) antibodies exemplify how the results in Fig. 4 were obtained for an A-type antibody (with *forward* cross-reactions) and a B-type antibody (without forward cross-reactions) respectively (see Experimental Procedures for details). There is a link in graph ***G*** connecting the IVI11C1 antibody with the IVI9C1 following the classification in Experimental Procedures, the deconvolution analysis predicts the absence of the IVI11C1 cognate immunogen (*Geobacter metallireducens* biomarkers) in BF2d (type I), and its absence but the existence of close related antigens from metal-reducer *Deltaproteobacteria* in BF1c, BF2b, BF2c, BF2e and BF2f extracts (type II). Only in BF1a and BF1b could the cognate immunogen of the IVI11C1 be present, though its positive experimental signal might also be due to related metal-reducer biomarkers (type III). As the IVI15C1 node does not point towards any other antibody node in graph ***G***, the deconvolution analysis predicts the absence of the IVI15C1 cognate immunogen in all transect extracts from both biofilms except in BF1c. In this transect, it is predicted that the cognate immunogen of the IVI15C1 or that of a related methanotroph *Gammaproteobacteria* are present in the sample (type III).

In summary the antibody graph and the deconvolution method confirmed the presence of cognate or closely related antigens of sulfur-oxidizers, aerobic heterotrophic *Alphaproteobacteria*, sulfur- and metal-reducing *Deltaproteobacteria*, metanotrophs, methanogens and members of the Ferritin superfamily (Fig. 4).

### Western-blot analysis confirmed the presence of specific proteins

**Figure 5:**
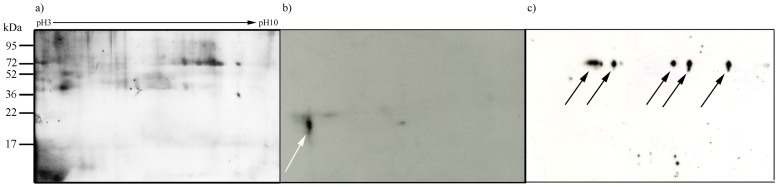
Western-blot analysis of BF2c extract. Proteins from BF2c extract were fractionated by two-dimensional gel electrophoresis on pH gradient-dried strips (pH 3–10). Replica gels were stained by silver-staining (a), electrotransferred to Immobilon-P membranes and then incubated with the PfuDPS antibody (anti-*Pyrococcus furiosus* DPS-protein) (b) or the IVI11C1 antibody (anti-*Geobacter sulfurreducens* cell extract) (c). Protein spots recognized by the PfuDPS or the IVI11C1 antibodies are marked by arrows.

To get insights about the biochemical compounds responsible for the immunoreactions obtained with EMChip66, a protein extract from BF2c transect sample was fractionated by two-dimensional electrophoresis, blotted and immunodetected with the IVI11C1 and the PfuDPS antibodies (Fig. 5). The PfuDPS (to a *Pyrococcus furiosus* DPS-protein) antibody recognized a protein spot of approximately 20 kDa and an isoelectric point (pI) of 4.0, which are similar to those of the cognate antigen [23]. Other protein spots were specifically recognized by the IVI11C1 antibody indicating that BF2c biofilm extract contained *G. sulfurreducens* related antigens, in agreement to the deconvolution results (see above).

### Molecular phylogenetic analyses support antibody microarray results

To corroborate and expand the results obtained with the immunoassays and deconvolution analyses, 16S rRNA gene sequencing and phylogenetic analyses were carried out with a transect sample from each biofilm (BF1c and BF2d). Different operational taxonomic units (OTU) were inferred and the closest relative within each OTU was shown in S3 Table. A total of 129 and 81 bacterial gene clones were sequenced from BF1c and BF2d, respectively. Two assemblies were tagged as chimera within the BF2d set. The rest of the sequences (127 and 81) clustered within 16 and 27 OTUs respectively, based on a 3% distance. Moreover, Chao1 richness index predicted 34 OTUs for BF1c (95% low and high confidence intervals between 29 and 58 OTUs) and 20 OTUs for BF2d (confidence intervals between 17 and 35 OTUs). These results were in agreement with the rarefaction analysis (S1 Fig.), which showed a significant difference between both samples. The curve for BF2d showed a high level of saturation compared to the curve for BF1c. This was reflected in the angle θ values (0.9 *vs* 1.6 respectively). The evenness of each sample shows also a significant difference according to the Simpson index, a value of 0.3 was obtained for the BF2c compared to 0.05 obtained for the BF1c.

**Figure 6:**
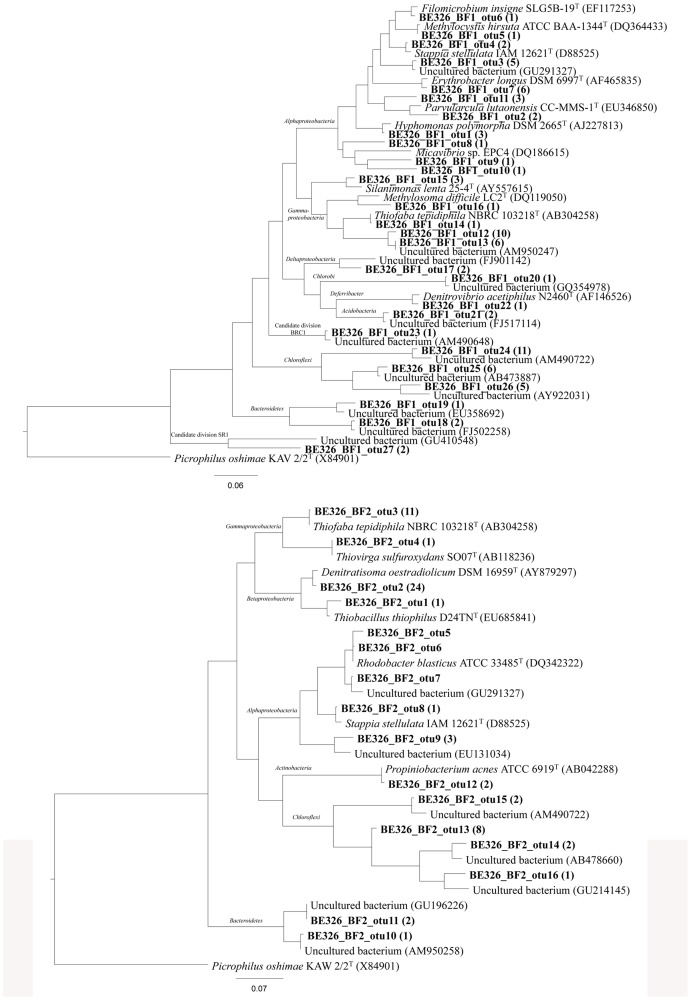
Phylogenetic affiliation of the 16S rRNA gene sequences retrieved from BF1c (upper part) and BF2d (bottom part) transect samples biofilms. A maximum-likelihood (PHYLML) phylogenetic tree was chosen as a consensus tree, after reconstructing the phylogeny by using different algorithms, substitution models and filters. The trees show the relationship between representative 16S rRNA gene clone sequences from BF1c and BF2d (in bold) and related strains and environmental clones from different bacterial phyla. The number of sequences grouped into that specific OTU is indicated in parentheses. Positional filters were applied to discard high variable positions and a total number of 490 and 570 columns respectively, were finally compared. Taxonomic classification according to Silva104 database is also shown. The scale bars represent 7 and 6% nucleotide substitutions per sequence position respectively.

The phylogenetic distribution of clones (Fig. 6) showed that members within *Proteobacteria* were major components in the analyzed libraries. Concerning to BF1c sample, several OTUs within the *Alphaproteobacteria* were related to marine genera of aerobic, mesophilic heterotrophic bacteria (*Parvularcula*, *Hyphomonas*, *Stappia*), some of them are known methano- and methylotrophs (*Filomicrobium*, *Methylocystis*) and one of them related to *Micavibrio* sp., that belonged to a genus of obligate epibiotic bacterial predators [24] . Also, the methanotrophic *Methylosoma* (*Gammaproteobacteria*) and some members within the *Halothiobacillaceae* family, likely involved in the sulfur cycle (e.g. *Thiofaba*) were detected. The only OTU detected within the *Deltaproteobacteria* was assigned to the *Myxococcales*, although distantly related to any cultured representative. It is also remarkable the OTU related to the marine, strict anaerobic denitrifier, *Denitrovibrio* (phylum *Deferribacter*). Finally it is also significant the amount of OTUs related to uncultured representatives within the *Chloroflexi* and *Bacteroidetes*, and some other candidate divisions. BF2d clone library retrieved a relatively high diversity with members from the *Alpha*-, *Beta*-, *Gamma*-, *Deltaproteobacteria*, *Actinobacteria*, *Chloroflexi*, *Bacteroidetes*, *Chlorobi*, *Deferribacter*, *Acidobacteria* and the candidate divisions BRC1 and SR1 were also detected. The OTUs within the *Betaproteobacteria* clustered into the families *Rhodocyclaceae* and *Hydrogeniphillaceae*. The second most abundant OTU from BF2d was closely related to the heterotrophic, nitrate-reducer, *Denitratisoma*. Within the family *Hydrogenophillaceae*, another OTU was related to sulfur-oxidizing genus *Thiobacillus.* All the OTUs within the class *Gammaproteobacteria*, clustered within the *Halothiobacillaceae* family and were related to the sulfur-oxidizers *Thiofaba* and *Thiovirga*. Several OTUs clustered within the *Alphaproteobacteria*. Most of them, including the most abundant OTU retrieved from this sample, clustered within the *Rhodobacteraceae* and were related to the genera *Rhodobacter* and *Stappia.* The rest of the OTUs retrieved from this sample were related to uncultured members within the phyla *Chloroflexi* and *Bacteroidetes*.

## Discussion

The microbial community composition and the metabolic potential of two biofilms collected from a deep South African gold mine has been analysed by complementary immunological and DNA sequencing techniques. Multiplex microarray immunoassays, together with a deconvolution method applied to the experimental data is a powerful tool to assign specific antigen antibody interactions and to identify the cognate immunogen in the samples. When comparing deconvolution and 16S rRNA phylogenetic analysis in one transect sample of each biofilm, both techniques coincided in detecting features related to aerobic sulfur-oxidizers, aerobic chemoorganotroph *Alphaproteobacteria* and metanotrophic *Gammaproteobacteria* (Fig. 4). In addition, EMChip66 detected immunological features from methanogens and sulfate-reducers whereas 16S rRNA gene sequencing detected phylotypes related to nitrate-reducers and anaerobic sulfur-oxidizers.

As a representative example, the deconvolution applied to IVE7C1 antibody confirmed that its immunological signal with BF2d extract (Fig. 3g) was not due to its cognate immunogen, *Halothiobacillus neapolitanus*, but to a very close relative. The analysis predicted that if *H. neapolitanus* were present, a positive with IVE3C1 deconvoluted signal should have been detected (Fig. 3g). By contrast, there was no positive fluorescence signal from the IVE7C1 antibody spot in BF1c (Fig. 3c). Those results were in agreement to those obtained by the 16S rRNA gene sequence analysis, which identified in BF2d clone library one OTU with 99% identity to *Thiofaba tepidiphila* and another OTU with 99% identity to *Thiovirga sulfuroxydans*, both of them belonging to the *Halothiobacillaceae* family. However, only one OTU with 99% identity to *Thiofaba tepidiphila* was detected in BF1c clone library. Those results suggested that those non-cognate antigens related to *Halothiobacillaceae* family and recognized by the IVE7C1 antibody could be below the sensitivity level of that antibody in the BF1c extract. As sulfur-oxidizer bacteria (SOB) represents a wide-ranging group of microorganisms from a phylogenetic point of view, the signals from the other antibodies related to sulfur-oxidizer cell cultures and environmental samples could come from well characterized bacteria from different taxonomic groups, e.g. *Thiobacillus thiophilus*, a sulfur-oxidizer belonging to *Betaproteobacteria*. It could also be possible that those signals might come from unidentified biosignatures of uncultured bacteria, e.g. from that related to AM950247 clone that was reported in an experimental bioreactor for sulfide oxidation [25]. It has been shown elsewhere [15], [16] that polyclonal antibodies recognized antigens of the exopolysaccharide fraction of microbial communities. It is not surprising that these antibodies recognized common immunological features in all biofilms that characterized them as sulfur-oxidizers since exopolysaccharides are involved in similar functions regardless their phylogenetic origin. One of these functions is the modification of microenvironments [26] by concentration of nutrients, in particular metal ions, as it has been shown by ICP-MS analysis of BF1c, BF2e and BF2f transect sample extracts (Table 2).

The deconvolution method also assured the detection of biosignatures from a mesophilic and heterotrophic *Alphaproteobacteria* related to *Acidiphillium* spp., the cognate immunogen of the IVG2C1 antibody, in BF1c and also in BF2e and BF2f transect samples. 16S rRNA sequences related to that bacterial group were more largely represented in BF1c clone library than in the BF2d one, in agreement to the immunoassay results. Moreover, one OTU related to *Erythrobacter* was identified in BF1c clone library. *Acidiphillium* and *Erythrobacter* are both *Alphaproteobacteria* and can be grouped as aerobic phototrophic bacteria, which could use bacteriochlorophyll for photosynthethic growth under aerobic conditions [27]. However, this metabolic process seemed very unlikely in the deep mine environment. Many of these sequences were related to marine *Alphaproteobacteria* genera (*Parvularcula*, *Hyphomonas*, *Stappia, Erythobacter*). In BF2d clone library two OTUs related to *Rhodobacter*, purple non-sulfur *Alphaproteobacteria*, were identified. Members of this genus can be found in marine and freshwater environments and can grow under microaerobic to aerobic conditions in the dark.

The deconvoluted positive signal of IVI15C1 antibody (raised against a *Methylomicrobium capsulatum* cell culture extract) in BF1c but not in BF2d biofilm extracts correlated with the phylogenetic analysis; 16S rRNA gene sequences very closely related to those from *Methylosoma difficile* were only detected in BF1c clone library (Fig. 4). Although direct methane measurements could not be carried out in water samples, the presence of dissolved methane flowing from boreholes in this area is not new [10]. Moreover, the detection of formaldehyde, as possible intermediate product of methane oxidation, could suggest an actual methane cycle supported by methanotroph and methanogenic microorganisms. In this sense, the deconvolution analysis confirmed the detection of *M. formicicum* or a close related methanogenic archaea associated to the IVJ4C1 positive signal in BF2d and BF2c samples (Figs. 1–4). Surprisingly, whereas archaeal rRNA clones have been reported from service and fracture water samples [2], [6], neither in the biofilms assayed in this work nor in those reported by Moser *et al*. [8] any archaeal PCR products were amplified.

Although *D. psychrophila* is a psychrophile, we interpret the positive signal with the anti- *D. psychrophila* antibody (IVF18C1) as a result of the presence of antigenic structures that can be common to sulfate reducer *Deltaproteobacteria* of the *Desulfobacterales* order or *Desulfobulbacea* family. We only obtained this positive in one of the samples (Fig. 1), indicating a specific niche with specific metabolic features. None of the other seven antibodies against different psychrophilic bacteria produced any positive signal. Two additional antibodies to *Deltaproteobacteria* strains (IVI10C1, to the sulfate-reducer *Desulfovibrio vulgaris*, and IVI11C1, to the metal-reducer *Geobacter sulfurreducens*) showed positive results in almost all the transect extracts. One OTU closely related to a *Deltaproteobacteria* was detected in BF1c clone library; although its metabolic characteristics cannot be extrapolated as its sequence did not show similarity to any characterized species. The PFLAs from Beatrix mine biofilms associated with sulfate- and metal-reducing bacteria were identified elsewhere [11]. Moreover, DNA sequences related to sulfate-reducing bacteria in fracture water of ultra-deep gold mines of South Africa were reported [28], [6]. Deconvolution analysis confirmed the presence of cognate antigens or very close related antigens to sulfur-reducer *Deltaproteobacteria* in BF1a and BF2b, BF2c, BF2d, BF2e and BF2f. The high Mn content measured by ICP-MS could be related to the existence of biosignatures closely related to metal-reducer *Deltaproteobacteria*, detected by the IVI11C1 antibody and inferred by the deconvolution analysis. Metal-reducing bacteria involved in metal cycling and in dissimilatory iron reduction are found in many different taxonomic groups as distant as *Acidobacteria* [29]; one OTU related to this bacterial group has been identified in BF1 clone library.

It is noteworthy that total Fe concentration in BF2e was 3 and 30 –fold higher than those in BF2c and BF1c, respectively. This fact correlates with the detection of PfuDPS and PfuFer antibody signals only in BF2e sample but neither in BF2c, BF1c, nor in the other samples. Only in BF2c out of the rest of the extracts the PfuDPS antibody signal could be detected. This last result was corroborated by Western-blot analysis but, unfortunately, there was no ICP analysis data from BF2c. PfuDPS and PfuFer antibodies are raised against the archaeal *Pyrococcus furiosus* proteins of the ferritin superfamily. The members of this superfamily are involved in iron metabolism; whereas ferritins are Fe^2+^ scavenging and storage proteins, DPS proteins are involved in Fe^2+^ detoxifying and DNA-protecting under starving conditions [30]. Therefore, the immunological detection of these proteins suggests a relevant metabolic activity related to iron in agreement with the iron accumulation in BF2e extract under the oligotrophic conditions of the fracture water that irrigated the biofilms.

**Figure 7:**
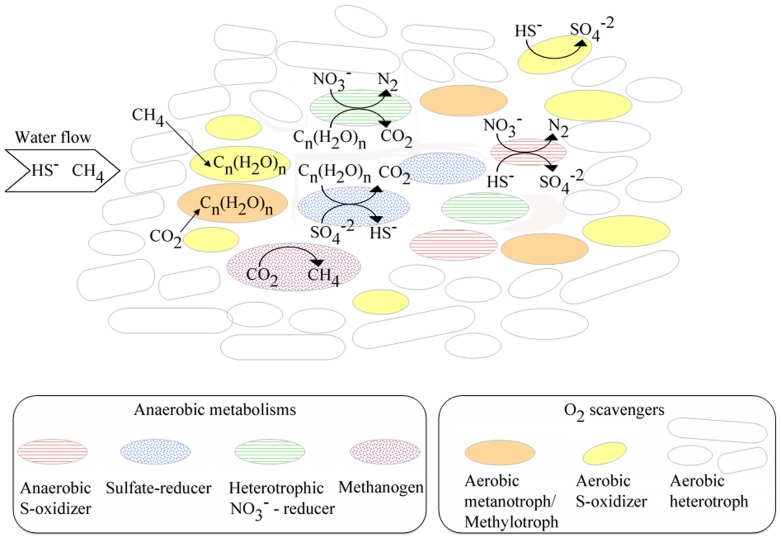
Syntrophic metabolisms in deep South African mine biofilms inferred from the complementary deconvolution method and the phylogenetic analysis. Metabolisms inferred from both methods are represented by solid circles (aerobic heterotrophs: white circles; aerobic S-oxidizers: yellow circles, and metanotrophs: orange circles), metabolisms inferred by deconvolution analysis by dotted circles (methanogens: purple circles and SRB: blue circles) and metabolisms inferred by 16S rRNA gene sequencing analysis are represented by horizontal lined circle (heterotrophic nitrate-reducers: green circle and anaerobic S-oxidizers: red circle).

In conclusion, we have demonstrated that EMChip66 antibody microarray can reveal new information about deep South African mine biofilms that escape to DNA sequencing. Altogether, the results indicated a microbial community in which a major group of primary producers are the sulfur-oxidizers (Fig. 7). Biosignatures and related sequences from members of the genera *Thiobacillus, Thiovirga*, *Thiofaba*, and other members within the *Halothiobacillaceae*, which have been detected by both type of analysis in transect samples of BF1 and BF2 biofilms, are frequent inhabitants in subsurface water in the deep mines [31]. Many of them are also known autotrophs [32], [33], which would contribute to the pool of available organic matter from the biomass decay or by releasing of soluble microbial products. Some other autotrophic population would include aerobic methanotrophs/methylotrophs detected by microarray immunoassay and confirmed by 16S rDNA sequencing analysis in a transect sample from BF1 (BF1c). Members of *Methylovulum* and *Methylocystis* genera have been previously detected in planktonic communities associated with fracture-derived groundwater in this area [34] where the concentrations of methane in the gas phase flowing from the boreholes are usually high [10]. Other heterotrophic bacteria, most of them belonging to the class *Alphaproteobacteria*, would be significant components of the biofilm external parts. Helping heterotrophic bacteria scavenging reactive oxygen species to decrease oxidative stress on chemolithotrophs have been found in other systems [35]. In this case, their main role is likely to keep the internal parts of the biofilm free of oxygen, to allow performing other anaerobic processes (e.g. denitrification and sulfate reduction). These heterotrophic bacteria might feed on the organic matter synthesized by the chemolithoautotrophs, many of which have been described to produce large amounts of exopolysaccarides [36]. According to this, the external layers of the biofilm would be mainly covered by aerobic-oxidizers. In this zone, sulphide removal might be achieved by aerobic sulphur-oxidizing bacteria. Sulphide removal in the internal parts of the biofilm can also be accomplished. Both sulphide and nitrate are abundant ions dissolved in the fissure water, which would be in agreement with a sulphur-oxidizing/denitrifying activity [10], [34]. Some chemolithoautotrophic bacteria related to *Thiobacillus*, can couple the oxidation of reduced sulphur compounds to the reduction of nitrate [37]. These internal low-oxygen layers would be an ecological niche for heterotrophic sulphate reducing bacteria (*Deltaproteobacteria*), metal-reducers (Mn and Fe), and nitrate-reducers (e.g. *Denitrovibrio*, *Denitratisoma*, *Stappia*). All these populations might feed on the organic compounds synthesized by the autotrophs or from cell lysates. Finally, while no archaeal sequences were obtained in this study, the immunoassays suggested that some methanogenic archaea might also be components of these communities. Although further experiments are needed to confirm this finding, the presence of methanogenic archaea in fracture-derived groundwater from this mine is not new [34] and it is compatible with indigenous microbial methanogenesis [38].

## Experimental Procedures

### Mine sites and biofilm sampling

We thank to Beatrix gold mine and Gold Fields Ltd. Company for given the permission to access the mine. All the procedures were done through the University of the Free State, Bloemfontein 9300, South Africa. Beatrix gold mine is located in the Witwatersrand Basin in the region of Welkom (South Africa). In this area, the quartzites from the Witwatersrand Supergroup and the basaltic succession of the Ventersdorp Supergroup are directly overlain by 400–800 m of Karoo sedimentary strata [10]. Occasional Ventersdorp and Karoo dikes cut all the strata and allow water-bearing fractures to occur within the contact between the dikes and the quartzites. The samples presented in this study were collected within the same corridor in Beatrix level 26, shaft 3 at 1.3 km below the surface. The two nearest boreholes with fracture-associated groundwater are located in the same corridor, with the following coordinates at the borehole end: X24012, Y19595, Z35.3 for BH1 and X24081, Y19621, Z36.4 for BH2. The coordinates are expressed in LO27 system and the Z value is elevation in meter above mean sea level (mamsl). Biofilms are located on the walls of the mine corridors, associated to water flows coming out through the fractures (Fig. 1). Vertical transect samples were taken directly from the bottom to the upper part of both biofilms into 50-mL Falcon tubes and transported to the laboratory of Free State University at Bloemfontein in an ice-chest in darkness conditions. Once in the laboratory, every transect sample was lyophilized and stored at −20°C until processing for extract preparation and DNA purification.

### Geochemical parameters measurements

Geochemical parameters of the fracture water from two boreholes flanking BF1 and BF2 biofilms were measured in field and in the lab. A multiparametric field probe was used to obtain *in situ* measurements of temperature (°C), pH and conductivity (mS cm^−1^). Readings were taken in triplicate and an average value was calculated later. Sulfide was also measured *in situ* by using Chemnet Kits. Water was collected into 15-mL centrifuge tubes (Falcon), filtered through 0.22 µm nylon filter (Acrodisk, Gelman). Filtered samples were collected for ion/cation and iron concentration measurements in the lab by ion chromatography. Total Organic Carbon (TOC), Dissolved Organic Carbon (DOC), and the concentration of phosphate, formaldehyde, and sulfide were done at the Institute of Ground Water Studies (University of the Free State, South Africa) using standard techniques as ion chromatography. Multi-elemental analysis from 30 mg of lyophilized transect biofilm samples were performed by ICP-MS (ELAN 9000, Perkin Elmer) at the Centro de Astrobiología (Madrid, Spain).

### Preparation of biofilm extracts

One gram of each lyophilized transect biofilm sample was resuspended in 20 mL of PBS (phosphate-buffered saline buffer), 0.2 M EDTA, 1x Roche protease inhibitor cocktail and then sonicated on ice by using a manual sonicator (Dr. Hielscher 50W DRH-UP50H sonicator, Hielscher Ultrasonics, Berlin, Germany) at 90% maximum power for 10 pulses of 30 seconds each, with 1 minute intervals on ice. The extracts were centrifuged at 10,000×g at 4°C for 15 min to remove cell debris. The supernatant was dialyzed thoroughly against ddH_2_O, frozen at −80°C, freeze-dried for 2 days into standard lyophilization equipment, and finally resuspended in PBS to obtain a protein final concentration of 10 mg mL^−1^.

### Determination of protein and total sugar concentrations

The protein concentrations of protein A-purified IgG and the lyophilized transect biofilm samples were quantified as described by Bradford [39] using IgG or BSA proteins, respectively, as standards. Total sugar content of biofilms was measured following the colorimetric method described by Dubois *et al.* [40] using glucose as standard.

### Antibody production, purification, labeling, and construction of EMChip66

The antibodies included in the environmental antibody microarray (EMChip66) were previously reported [21]. These 66 antibodies (S2 Table) were produced against extracts from natural samples, cell cultures and some purified proteins. They can detect specific biosignatures from sulfur-oxidizer cell cultures and sulfur-rich environments (antibodies 1–15; S2 Table), psychrophilic bacteria (17–23), halophilic microorganisms (41–43, 52 and 58–59), sulfate-reducer (16 and 31) and metal-reducer bacteria (11–12), and from other different group-specific bacteria and archaea. We included antibodies to psychrophiles because EMChip66 is a demonstration of the multiplex detection of microbial strains and metabolites from different environments. We included in the chip microbes and biomarkers from extreme niches and environments because the aim is to use it for monitoring a variety of habitats. Regardless the low temperatures, Psychrophiles include very wide range of phylogenetic groups and metabolisms. Alternatively, if low temperature is a limiting factor, no positive were expected in the samples studied in this work. All the antibodies were purified by Protein A affinity chromatography and fluorescently labeled as reported previously in [41], [14]. The EMChip66 slides were constructed as described [15]. Briefly, (i) we used a commercial protein printing buffer 2x (Whatman, Schleicher & Schuell, Sandford, ME) and 0.02% Tween 20 as the spotting solution; (ii) printing was done in a duplicate spot-pattern on epoxy-activated glass slides (Arrayit Corp., Sunnyvale, CA) with a MicroGrid II TAS arrayer (Biorobotics, Genomic Solutions, UK). Up to 9 different arrays containing a duplicate spot pattern for the 66 antibodies and their corresponding protein A-purified pre-immune serum were spotted on a microscope slide so that each microarray fits with one of the nine flow cells in a multi array analysis module (MAAM) device [15], [42].

### Sandwich microarray immunoassay (SMI) procedure

Each slide was blocked by incubating for ten minutes in 5% (w/v) BSA in 0.5 M Tris-HCl pH 9 and then in 2% (w/v) BSA in 0.5 M Tris-HCl pH 8, for 30 minutes with gentle agitation at room temperature. After drying the chip by short centrifugation, the slide was set up into the MAAM device and 50 µL of the biofilm crude extract in TBSTRR (0.4 M Tris-HCl pH 8, 0.3 M NaCl, 0.1% Tween 20) were inoculated into one of the nine flow cells to flood the EMChip66. Different amounts of transect extracts, between 10 and 100 ng of total protein for each sample regarding their protein content, were assayed. After 1 h of incubation at room temperature, the flow cells were washed with TBSTRR and the immunoassay revealed by 1 h incubation with a mixture of 66 different fluorescent antibodies, as described elsewhere [21]. The replicate average values lower than background were discarded [21]. Then, their data were transformed into a matrix (1 for values higher than background and 0 for those lower than background). This matrix was unsupervised hierarchically clustered and visualized as a heat map using Cluster 2.11 and TreeView 1.60 software [43].

### Antibody graph associated to an antibody microarray and deconvolution analysis of sandwich microarray immunoassays

The deconvolution method applied and extended in this work was presented in [21]. Its target is to characterize an experimental sample by disentangling the cross-reactivity inherent to the antibody sandwich microarray format. In this section we briefly explain the procedure to apply it (see original article for details), but we also explore and analyze thoroughly many of its strengths and limitations when characterizing open systems, a question only sketched in [21].

The method is based on the information contained in an antibody graph. A weighted and directed antibody graph 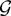 with *N* nodes and *l* links and its associated matrix ***G*** of size *N×N* can be assigned to an antibody microarray in sandwich format. *N* is the number of antibodies represented in the microarray and *l* the number of positive elements *G_ij_* of the matrix ***G***, being *G_ij_* the extent of cross-reactivity of two antibodies *i* and *j* referred to the cognate immunogen of the tracer antibody *j*. Matrix ***G*** and graph 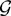 are related as follows. Every positive *G_mn_* represents a *forward cross-reaction* between the tracer antibody *n* and the printed antibody *m*, and is plotted as one link that starts in node *n*, points towards node *m* and whose weight is *G_mn_* (see Rivas *et al*., (2011) for instructions to obtain *G_mn_*). Note that all nodes have a self-loop of weight *G_jj_*  =  1. As the antibody microarray used in this paper is the same as the one studied in [21], the matrix ***G*** and the antibody graph 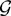 are also the same (see Fig. 2 for the plot of the antibody graph).

The method to deconvolute antibody cross-reactivities in the sandwich immunoassay format relies on the fact that the fluorescence intensity of one antibody spot on the microarray can be approximated as the sum of the contributions of all the antibodies that cross-react with it (i.e., that point towards it in the antibody graph, including itself). This approximation is valid when the antibody is in the regime in which the relation between amount of antigen and fluorescence is approximately linear and far away from saturation. Consequently, the problem can be expressed as a set of *N* linear equations, which in matrix terminology becomes ***F*** *=* G·F^*^, where ***G*** is the matrix associated to the graph 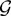, and ***F*** and F^*^ are column vectors that represent the fluorescence intensity measured in each spot and the deconvoluted signals (which in fact represent the unknown fluorescence intensity of each antibody due to its cognate immunogen), respectively. The solution of the problem becomes F^*^≈***G^-1^·F.*** The experimental noise associated to the measurement of ***F*** was treated as in [21]. By analyzing the experimental measurements ***F*** and the deconvoluted signals F^*^, the deconvolution method assures the existence or absence of a certain antigen when treating with closed systems, that is, samples containing antigens whose cognate antibodies are present in the microarray. However, in this work we study an open system, because *a priori* we do not know whether the antigens present in the experimental sample will have their cognate antibodies in the microarray or not. In order to summarize the information that the deconvolution method can obtain from the nature of an open system, let us call *F_j_* the fluorescence intensity of spot *j* and F^*^_j_ its deconvoluted signal (which represents an approximation to the fraction of the fluorescence intensity of antibody *j* that is due to its cognate immunogen). We classify the antibodies in two groups: antibodies that forward cross-react with at least one antibody of the microarray (type-A), and antibodies that do not show any forward cross-reaction (type-B).

#### Type-A antibodies

Let us suppose that there is a link in graph ***G*** connecting antibody *j* and antibody *i* (that is, *G_ij_*>0). Then, four main situations are possible for the cognate antigen of antibody *j*: (I) *F_j_*≈0: Antigen *j* is not present in the sample; (II.a) *F_j_>>*0 and F^*^_j_≤0: Antigen *j* is not present in the sample, and its positive experimental signal is due to the existence of a related antigen whose cognate antibody can be -or not- represented in the microarray; (II.b) *F_j_>>*0, F^*^_j_>>0, and F^*^_i_<<0: antigen *j* is not present in the sample, and its positive experimental signal is due to the existence of a related antigen whose cognate antibody is not in the microarray -and therefore is unknown-; and (III) *F_j_>>*0, F^*^_j_>>0 and F^*^_i_ ≥0: Either antigen *j* or a close related antigen is present in the sample.

#### Type-B antibodies

The second type of antibody is the case in which there is not a link in graph ***G*** connecting antibody *j* with any other antibody *i* (that is *G_ij_* = 0 for all *i*). For this situation, the possibilities are the following: (I) *F_j_*≈0: Antigen is not present in the sample; (II) *F_j_>>*0 and F^*^_j_≤0: Antigen *j* is not present in the sample, and its positive experimental signal is due to the existence of a related antigen whose cognate antibody can be or not represented in the microarray; and (III) *F_j_>>*0 and F^*^_j_>>0: Either antigen *j* or a close related antigen is present in the sample.

As shown, the existence of forward cross-reactivity events allows obtaining for type-A antibodies more detailed information than for type-B antibodies. In summary, for each antibody represented in the microarray, independently of its type, we can organize the information obtained applying the deconvolution method in the following three different possibilities: (I) Its cognate antigen is not present in the sample; (II) Its cognate antigen is not present in the sample, but a related antigen is; and (III) either its cognate antigen or a close related antigen is present in the sample.

### DNA extraction and PCR amplification

DNA was extracted from 1 g of each lyophilized transect biofilm sample with MoBio DNA extraction kit according to the manufacturer's instructions (MoBio laboratories, Inc.). Total genomic DNA was extracted in triplicate for each sample. This DNA was used as template for 16S rRNA genes PCR amplification with the bacterial universal primers 16SF as forward (positions 8-27 of *E. coli* 16S rRNA, 5′-AGAGTTTAGTCATGGCTCA) and 16SR as reverse (positions 1057-1074, 5′-CACGAGCTGACGACAGCCG) for 16S rRNA gene; and 23SF (positions 105-127; 5′ GCGATTTCYGAAYGGGGRAACCC) and 2241R (positions 2236-2253; 5′ ACCGCCCCAGTHAAACT) for *E. coli* 23S rRNA gene. PCR conditions were as follows: 95°C 5 min; 30 cycles of 95°C 20 s, 56°C 30 s, 72°C 2 min; 72°C 10 min; 4°C. In addition, amplification of 16S rRNA archaeal genes was carried out by using two different pairs of primers: 21F (5′ TTCCGGTTGAGCCGGA 3′) with 958R (5′ YCCGGCGTTGCCAATT 3′) and 1AF (5′ TCYGKTTGATCCYGSCRGAG 3′) with 1100R (5′TGGGTCTCGCTCGTT 3′). In this case attempts for amplifying archaeal PCR products were unsuccessful despite amplifications variation in different annealing temperature and DNA concentrations. Bacterial amplicons were purified with Qiagen PCR purification kit columns (QIAGEN) and cloned into TOPO 3.1 vector (Invitrogen) using TOPO TA cloning kit, according to the manufacturer's instructions. Transformed colonies were sent for sequencing within the Center of Astrobiology (Madrid, Spain) sequencing facility. Clones were sequenced from both ends with M13F and M13R primers.

### Phylogenetic analysis of the bacterial 16S rRNA gene sequences

Partial length 16S rRNA gene sequences were obtained by assembling both partial sequences. NAST-aligned sequences were checked for chimerical assemblies with the sequence utility ChimeraSlayer [44]. Non-chimeric full-length sequences were imported into the Arb phylogenetic package [45]. The Silva SSU_Ref_104 database [46] was previously updated by including new BLASTn hits obtained from our sequences. Multiple alignments were manually corrected by using the editing tool in the software and sequences added into a stable guide tree. A distance matrix was obtained with the algorithm neighbour joining using a Felsenstein correction for DNA. The matrix was used as an input file with mothur version 1.17.0 [47]. The default algorithm furthest neighbour was chosen for cluster analysis. Rarefaction curves (1,000 resampling) and a variety of community richness calculators (Chao1) and diversity indexes (Simpson) implemented in mothur were obtained. The estimated steepness values (angle θ) of the line tangent to the rarefaction were also determined by using the last point. The community structure was also analyzed in order to study the sharing level between the different samples and sites. Phylogenetic reconstruction was obtained by using the different algorithms Arb neighbour joining, PHYLIP DNAPars (Parsimony version 3.6a3) and PHYLML (maximum likelihood version 2.4.5) implemented in the Arb software. Only sequences >900 nucleotides were used. Filters excluding the most variable positions were also employed. A consensus tree was selected among the generated trees by comparing the stability of the branching. Only one representative of each OTU was finally kept on the tree for better understanding.

Sequences of unique phylotypes found in this study have been deposited on GenBank under accession numbers JX298417- JX298443 and JX298444- JX298459 for BF1c and BF2d transect samples from BF1 and BF2 Beatrix gold mine biofilms, respectively.

### Two-dimensional gel electrophoresis

150 µg of proteins were precipitated from a BF2d extract sample by 2-D Clean-Up kit (GE Healthcare) and then fractionated by two-dimensional electrophoresis (2DE) on an Immobiline DryStrip gradient (pH 3–10; 7 cm; Amersham Biosciences, Uppsala, Sweden) as described [48]. Isoelectric focusing was performed in IPGPhor-I (Amersham Biosciences, Uppsala, Sweden), to a final value of 8000 Vh. SDS-PAGE in the second dimension was performed in 15% polyacrylamide gels. After electrophoresis, the gels were stained with silver staining with Plus One Silver Staining Kit (GE Healthcare) or electrotransferred (transfer buffer: 25 mM Tris, 192 mM glycine, pH 8.3) to polyvinylidene difluoride membranes (Immmobilon P, Amersham Biosciences) for Western-blot analysis, as described below.

### Western-blot analysis

After blocking with 5% bovine serum albumin (BSA) (w/v) in PBS buffer, proteins electrotransferred to polyvinylidene difluoride membranes were incubated for 1 h at room temperature with 20 µg mL^−1^ of the appropriated diluted protein A-purified antibody in PBST (PBS buffer, 0.1% Tween 20) and washed with PBST. Then, immunoblots were treated with goat anti-rabbit IgG peroxidase-conjugated antibody (1:10,000 dilution, 1 h) using the enhanced chemiluminescence (ECL) Western blotting detection system kit (GE Healthcare). The blots were exposed to Kodak X-Omat LS Film (Kodak) at room temperature.

## Supporting Information

S1 Fig **Rarefaction analysis from BF1c and BF2d clone libraries.**
(DOCX)

S1 Table **Protein and total sugar content of transect sample extracts.**
(DOCX)

S2 Table **Antibodies used for the immunoassays and the immunogen used to produce them.**
(DOC)

S3 Table **Closest BLASTn relative of representative 16S rRNA gene clones within each OTU, retrieved from BF1c and BF2d transect samples from BF1 and BF2 Beatrix gold mine biofilms, respectively.** A cultured representative was selected when available.
(DOCX)
