# Supplementary figures and images for: Deciphering the Prokaryotic Community and Metabolisms in South African Deep-Mine Biofilms through Antibody Microarrays and Graph Theory
Source: PLoS One. 2014 Dec 22;9(12):e114180. doi: 10.1371/journal.pone.0114180 (PMC4273990; doi:10.1371/journal.pone.0114180)

**Figure S1**. **Rarefaction analysis from BF1c and BF2d clone libraries.**


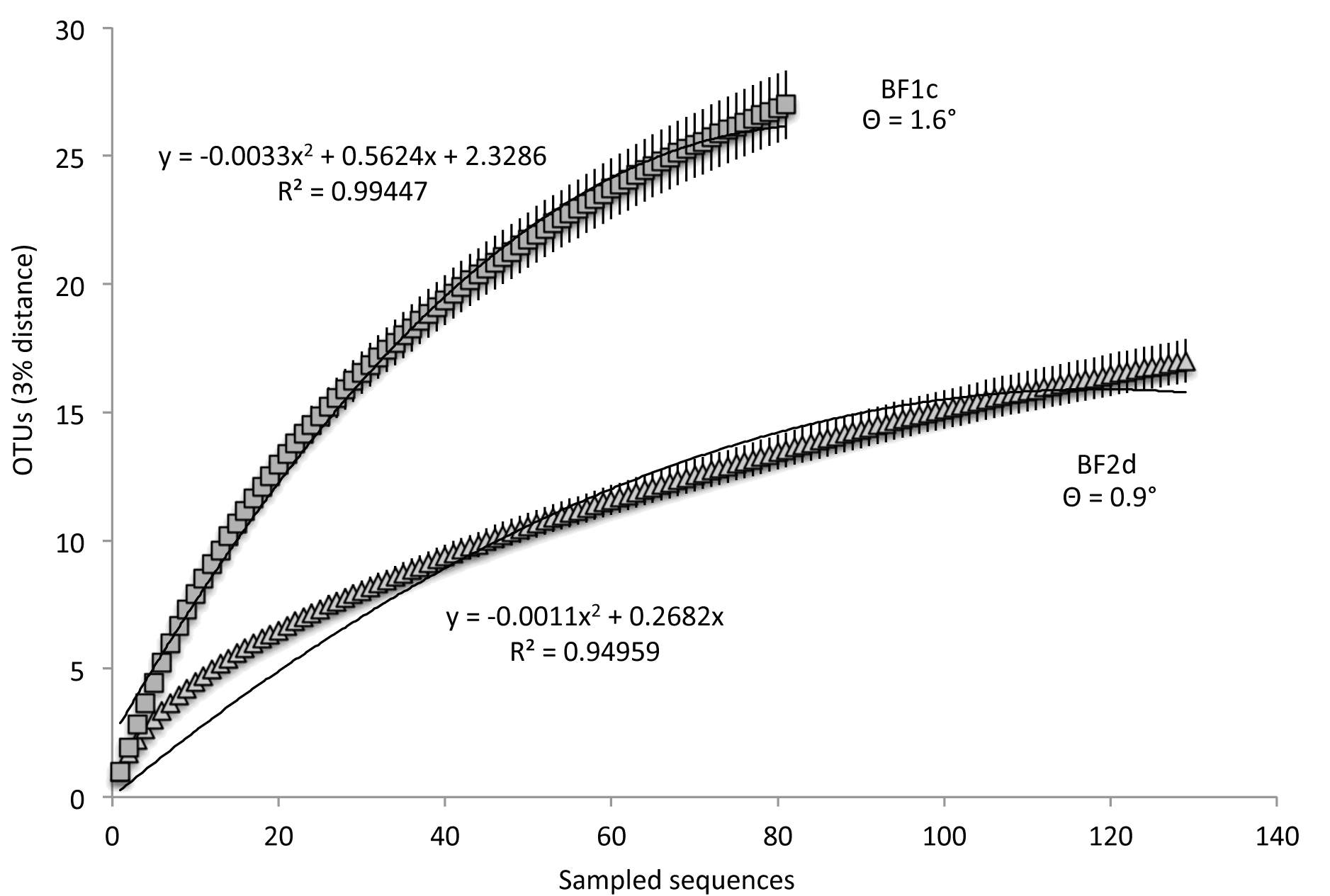

Supplement: S1 Fig — Rarefaction analysis from BF1c and BF2d clone libraries. (DOCX) [file pone.0114180.s001.docx]
