# Supplementary material for: Deciphering the Prokaryotic Community and Metabolisms in South African Deep-Mine Biofilms through Antibody Microarrays and Graph Theory
Source: PLoS One. 2014 Dec 22;9(12):e114180. doi: 10.1371/journal.pone.0114180 (PMC4273990; doi:10.1371/journal.pone.0114180)
Supplement: S1 Table — Protein and total sugar content of transect sample extracts. (DOCX) [file pone.0114180.s002.docx]

| **Table S1. Protein and total sugar content of transect sample extracts.** | | |
| --- | --- | --- |
|  | **mg protein g^-1^ of dry weight biofilm** | **mg total sugars g^-1^ of dry weight biofilm** |
| **BF1a** | 0.302 | 0.689 |
| **BF1b** | 0.185 | 0.314 |
| **BF1c** | 0.15 | 0.245 |
| **BF2a** | 0.67 | 1.156 |
| **BF2b** | 0.322 | 1.168 |
| **BF2c** | 0.439 | 0.865 |
| **BF2d** | 0.369 | 0.755 |
| **BF2e** | 0.285 | 0.41 |
| **BF2f** | 0.228 | 0.454 |
