# Supplementary material for: Deciphering the Prokaryotic Community and Metabolisms in South African Deep-Mine Biofilms through Antibody Microarrays and Graph Theory
Source: PLoS One. 2014 Dec 22;9(12):e114180. doi: 10.1371/journal.pone.0114180 (PMC4273990; doi:10.1371/journal.pone.0114180)
Supplement: S2 Table — Antibodies used for the immunoassays and the immunogen used to produce them. (DOC) [file pone.0114180.s003.doc]

| **Table S2**. Antibodies used for the immunoassays and the immunogen used to produce them. | | | | | |
| --- | --- | --- | --- | --- | --- |
| **Ab number** | **Ab name** | **Source of immunogen** | **Sample/ Culture conditions** | **Immunogen / Fraction** | **Reference** |
| 1 | A139 | *Leptospirillum ferrooxidans* | Batch (N_2_ fixing) | Sonicated cells | Parro et al., 2005 |
| 2 | A184 | *Acidithiobacillus thiooxidans* | Batch + S | Whole cells (intact + sonicated) | Parro et al., 2005 |
| 3 | IA2C1 | Río Tinto (3.1 stream) | Green filaments | Cellular fraction | Rivas et al., 2008 |
| 4 | IA3C1 | Río Tinto (3.1 stream) | Black filaments | Cellular fraction | Rivas et al., 2008 |
| 5 | IC1C1 | Río Tinto (3.2 water dam) | Sediments | Cellular fraction | Rivas et al., 2008 |
| 6 | IC3C3 | Río Tinto (Main spring) | Brown filaments | Cellular fraction | Rivas et al., 2008 |
| 7 | IC4C1 | Río Tinto (3.2 water dam) | Brown filaments | Cellular fraction | Rivas et al., 2008 |
| 8 | IC6C1 | Río Tinto (3.1 water dam) | Red sediment 1-2 cm under crust | Cellular fraction | Rivas et al., 2008 |
| 9 | IC7C1 | Río Tinto (3.2 water dam) | Dried wall sediments | Cellular fraction | Rivas et al., 2008 |
| 10 | ID4S2 | Peña de Hierro (154 m deep) | 4-61a sample (MARTE project) | Supernatant from EDTA wash | Rivas et al., 2008 |
| 11 | IVE1C1 | *Leptospirillum ferrifillum* (LPH2) | Fermentor | Whole cells | Rivas et al., 2008 |
| 12 | IVE2C1 | *Leptospirillum ferrifillum* spp. | Batch + Fe^2+^ | Whole cells | Rivas et al., 2008 |
| 13 | IVE3C1 | *Acidithiobacillus ferrooxidans* | Batch + Fe^2+^ | Whole cells | Rivas et al., 2008 |
| 14 | IVE5C1 | *Acidithiobacillus albertensis* | Batch + S | Whole cells | Rivas et al., 2008 |
| 15 | IVE7C1 | *Halothiobacillus neapolitanus* DSM 16832 | Batch + S | Whole cells | Rivas et al., 2011 |
| 16 | IVF18C1 | *Desulfotalea psychrophila* | Biomass from DSM 12343 | Whole cells | Rivas et al., 2011 |
| 17 | IVF2C1 | *Shewanella gelidimarina* ATCC® 700752 | Batch (Marine broth 4ºC) | Whole cells | Rivas et al., 2008 |
| 18 | IVF2S2 | *S. gelidimarina* ATCC® 700752 | Batch (Marine broth 4ºC) | Supernatant from EDTA wash | Rivas et al., 2008 |
| 19 | IVF4C1 | *Psychrobacter burtonensis* ATCC® 700359 | Batch (Marine broth 4ºC) | Whole cells | Rivas et al., 2008 |
| 20 | IVF4S2 | *Psychrobacter burtonensis* ATCC® 700359 | Batch (Marine broth 4ºC) | Supernatant from EDTA wash | Rivas et al., 2008 |
| 21 | IVF5C1 | *Psychrobacter frigidicola* ATCC® 700361 | Batch (Harpo´s medium 15ºC) | Whole cells | Rivas et al., 2008 |
| 22 | IVF6C1 | *Cryobacterium psychrophilum* DSM 4854 | Batch (TSA 4ºC) | Whole cells | Rivas et al., 2008 |
| 23 | IVF7C1 | *Colwellia psychrerythraea* DSM 8813 | Batch (Bacto Marine Broth 4ºC) | Whole cells | Rivas et al., 2011 |
| 24 | IVG1C1 | *Acidocella aminolytica* DSM 11237 | Batch | Whole cells | Rivas et al., 2011 |
| 25 | IVG2C1 | *Acidiphillium* sp. | Batch | Whole cells | Rivas et al., 2011 |
| 26 | IVG3C1 | *Acidobacterium capsulatum* DSM 11244 | Batch | Whole cells | Rivas et al., 2011 |
| 27 | IVG4C1 | *Thermus scotoductus* | Batch | Whole cells | Rivas et al., 2011 |
| 28 | IVG4C2 | *Thermus scotoductus* | Batch | Insoluble cell pellet from S100 | Rivas et al., 2011 |
| 29 | IVG5C1 | *Sulfobacillus acidophilus* | Biomass from DSM No 10332 | Whole cells | Rivas et al., 2011 |
| 30 | IVH1C1 | *Bacillus subtilis* 168 (spores) | Batch (Schaeffer medium) | Whole spores | Fernández-Calvo et al., 2006 |
| 31 | IVI10C1 | *Desulfovibrio vulgaris* | Biomass from DSM No 644 | Whole cells | Rivas et al., 2008 |
| 32 | IVI11C1 | *Geobacter sulfurreducens* | Biomass from DSM No 12127 | Whole cells | Rivas et al., 2008 |
| 33 | IVI12C1 | *Geobacter metallireducens* | Biomass from DSM No 7210 | Whole cells | Rivas et al., 2008 |
| 34 | IVI13C1 | *Thermotoga maritima* | Biomass from DSM No 3109 | Whole cells | Rivas et al., 2008 |
| 35 | IVI14C1 | *Verrucomicrobium spinosum* | Biomass from DSM No 4136 | Whole cells | Rivas et al., 2008 |
| 36 | IVI15C1 | *Methylomicrobium capsulatum* | Biomass from DSM No 6130 | Whole cells | Rivas et al., 2008 |
| 37 | IVI16C1 | *Planctomyces limnophilus* | Biomass from DSM No 3776 | Whole cells | Rivas et al., 2008 |
| 38 | IVI17C1 | *Hydrogenobacter thermophilus* | Biomass from DSM No 6534 | Whole cells | Rivas et al., 2008 |
| 39 | IVI19C1 | *Desulfosporosinus meridiei* | Biomass from DSM No 13257 | Whole cells | Rivas et al., 2011 |
| 40 | IVI1C1 | *Pseudomonas putida* | Batch (LB) | Whole cells | Rivas et al., 2008 |
| 41 | IVI20C1 | *Salinibacter ruber* M8 | Batch | Whole cells | Rivas et al., 2011 |
| 42 | IVI21C1 | *Salinibacter ruber PR1* | Batch | Whole cells | Rivas et al., 2011 |
| 43 | IVI21C2 | *Salinibacter* ruber PR2 | Batch | Whole cells | Rivas et al., 2011 |
| 44 | IVI2C1 | *Bacillus* spp. (environ. isolate)* | Batch (LB) | Whole cells | Rivas et al., 2008 |
| 45 | IVI3C1 | *Shewanella oneidensis* | Batch (LB) | Whole cells | Rivas et al., 2008 |
| 46 | IVI4C1 | *Burkholderia fungorum* | Batch (LB) | Whole cells | Rivas et al., 2008 |
| 47 | IVI5C1 | *S. oneidensis* | Anaerobic (fumarate) | Whole cells | Rivas et al., 2008 |
| 48 | IVI6C3 | *Azotobacter vinelandii* | Batch culture (LB) | EDTA washed cells (sonicated) | Rivas et al., 2008 |
| 49 | IVI7C1 | *Bacillus* spp. (environmental isolate)* | Batch culture (LB) | Whole cells | Rivas et al., 2008 |
| 50 | IVI8C1 | *B. subtilis* 3610 | Biofilm | Whole cells | Rivas et al., 2008 |
| 51 | IVI9C1 | *Deinococcus radiodurans* | Biomass from DSM No 20539 | Whole cells | Rivas et al., 2008 |
| 52 | IVJ1C1 | *Haloferax mediterranei* | Batch culture | Whole cells | Rivas et al., 2008 |
| 53 | IVJ2C1 | *Methanococcoides burtonii* | Biomass from DSM No 6242 | Whole cells | Rivas et al., 2008 |
| 54 | IVJ3C1 | *Thermoplasma acidophilum* | Biomass from DSM No 1728 | Whole cells | Rivas et al., 2008 |
| 55 | IVJ4C1 | *Methanobacterium formicicum* | Biomass from DSM No 1535 | Whole cells | Rivas et al., 2008 |
| 56 | IVJ5C1 | *Methanosarcina mazeii* | Biomass from DSM No 3647 | Whole cells | Rivas et al., 2008 |
| 57 | IVJ6C1 | *Pyrococcus furiosus* | Biomass from DSM No 3638 | Whole cells | Rivas et al., 2011 |
| 58 | IVJ8C1 | *Halorubrum* sp. | Batch | Whole cells | Rivas et al., 2011 |
| 59 | IVJ9C1 | *Halobacterium* sp. | Batch | Whole cells | Rivas et al., 2011 |
| 60 | BaFER | Bacterio ferritin | Bacterial ferritin protein | Purified protein | Rivas et al., 2011 |
| 61 | PfuDPS | *Pyrococcus furiosus* | DPS-like protein | Purified protein | Rivas et al., 2011 |
| 62 | PfuFER | *Pyrococcus furiosus* | Ferritin protein | Purified protein | Rivas et al., 2011 |
| 63 | SsoDPS | *Sulfolobus solfataricus* | DPS-like protein | Purified protein | Rivas et al., 2011 |
| 64 | TscABCt | *Thermus scotoductus* | ABC transporter protein | Purified protein | Rivas et al., 2011 |
| 65 | TscCrR | *Thermus scotoductus* | Chromate reductase protein | Purified protein | Rivas et al., 2008 |
| 66 | TscFeR | *Thermus scotoductus* | Iron reductase protein | Purified protein | Rivas et al., 2008 |

Rivas LA, García-Villadangos M, Moreno-Paz M, Cruz-Gil P, Gómez-Elvira J, et al. (2008) A 200-antibody microarray biochip for environmental monitoring: searching for universal microbial biomarkers through immunoprofiling. Anal Chem 80: 7970-7979.

Rivas LA, Aguirre J, Blanco Y, González-Toril E, Parro V (2011) Graph-based deconvolution analysis of multiplex sandwich microarray immunoassays: applications for environmental monitoring. Environ Microbiol 13: 1421-1432.

Parro V, Rodríguez-Manfredi JA, Briones C, Compostizo C, Herrero PL, et al. (2005) Instrument development to search for biomarkers on Mars: terrestrial acidophile iron-powered chemolithoautotrophic communities as model systems. Planet Space Sci 53: 729-737.

Fernández-Calvo, P., Luis A. Rivas, Christian Näke, Miriam García-Villadangos, Javier Gómez-Elvira, and Víctor Parro (2006) A multi-array competitive immunoassay for the detection of broad-range molecular size organic compounds relevant for astrobiology. *Planetary and Space Science* 54: 1612-1621.
