# Supplementary material for: Deciphering the Prokaryotic Community and Metabolisms in South African Deep-Mine Biofilms through Antibody Microarrays and Graph Theory
Source: PLoS One. 2014 Dec 22;9(12):e114180. doi: 10.1371/journal.pone.0114180 (PMC4273990; doi:10.1371/journal.pone.0114180)
Supplement: S3 Table — Closest BLASTn relative of representative 16S rRNA gene clones within each OTU, retrieved from BF1c and BF2d transect samples from BF1 and BF2 Beatrix gold mine biofilms, respectively. A cultured representative was selected when available. (DOCX) [file pone.0114180.s004.docx]

| **Table S3.** Closest BLASTn relative of representative 16S rRNA gene clones within each OTU, retrieved from BF1c and BF2d transect samples from BF1 and BF2 Beatrix gold mine biofilms, respectively. A cultured representative was selected when available. | | | |
| --- | --- | --- | --- |
| **OTU** | **BLAST hit** | **Access #** | **% Identity** |
| BE326_BF1_otu1 | *Hyphomonas* sp. MOLA 55 | [AM990830](http://www.ncbi.nlm.nih.gov/nucleotide/187319394?report=genbank&log$=nuclalign&blast_rank=12&RID=V563SH7F015) | 1242/1270 (98%) |
| BE326_BF1_otu2 | Uncultured bacterium | [AF234706](http://www.ncbi.nlm.nih.gov/nucleotide/13171264?report=genbank&log$=nuclalign&blast_rank=1&RID=V563SH7F015) | 1237/1270 (97%) |
| BE326_BF1_otu3 | *Rhodobacter vinaykumaraii* JAJA249 | [AM600642](http://www.ncbi.nlm.nih.gov/nucleotide/138753490?report=genbank&log$=nuclalign&blast_rank=2&RID=V563SH7F015) | 1233/1276 (97%) |
| BE326_BF1_otu4 | *Stappia* sp. F2 | [JF899875](http://www.ncbi.nlm.nih.gov/nucleotide/335893105?report=genbank&log$=nuclalign&blast_rank=1&RID=V563SH7F015) | 1290/1292 (99%) |
| BE326_BF1_otu5 | *Methylocystis* sp. M42/1 | [AJ458499](http://www.ncbi.nlm.nih.gov/nucleotide/21685000?report=genbank&log$=nuclalign&blast_rank=1&RID=V563SH7F015) | 996/1000 (99%) |
| BE326_BF1_otu6 | *Filomicrobium* sp. 4-54 | [HM467169](http://www.ncbi.nlm.nih.gov/nucleotide/301071083?report=genbank&log$=nuclalign&blast_rank=2&RID=V563SH7F015) | 953/977 (98%) |
| BE326_BF1_otu7 | *Erythrobacter* sp. JL-475 | [DQ104409](http://www.ncbi.nlm.nih.gov/nucleotide/73695911?report=genbank&log$=nuclalign&blast_rank=1&RID=V563SH7F015) | 1279/1296 (99%) |
| BE326_BF1_otu8 | Uncultured bacterium | [GQ115967](http://www.ncbi.nlm.nih.gov/nucleotide/238352144?report=genbank&log$=nuclalign&blast_rank=1&RID=V563SH7F015) | 1170/1294 (90%) |
| BE326_BF1_otu9 | Uncultured bacterium | [FJ792144](http://www.ncbi.nlm.nih.gov/nucleotide/225675148?report=genbank&log$=nuclalign&blast_rank=1&RID=V563SH7F015) | 919/983 (93%) |
| BE326_BF1_otu10 | Uncultured bacterium | [AB473970](http://www.ncbi.nlm.nih.gov/nucleotide/218774992?report=genbank&log$=nuclalign&blast_rank=1&RID=V563SH7F015) | 912/949 (96%) |
| BE326_BF1_otu11 | Bacterium dMB-MAT32 | [AB458525](http://www.ncbi.nlm.nih.gov/nucleotide/269993879?report=genbank&log$=nuclalign&blast_rank=5&RID=V5CCX5PF015) | 1228/1320 (93%) |
| BE326_BF1_otu12 | Uncultured bacterium | [GQ355028](http://www.ncbi.nlm.nih.gov/nucleotide/256956226?report=genbank&log$=nuclalign&blast_rank=1&RID=V5CCX5PF015) | 1289/1342 (96%) |
| BE326_BF1_otu13 | Uncultured bacterium | [EU662563](http://www.ncbi.nlm.nih.gov/nucleotide/194478087?report=genbank&log$=nuclalign&blast_rank=1&RID=V5CCX5PF015) | 1334/1342 (99%) |
| BE326_BF1_otu14 | *Halothiobacillus* sp. WJ18 | [AY096035](http://www.ncbi.nlm.nih.gov/nucleotide/20750395?report=genbank&log$=nuclalign&blast_rank=1&RID=V5CCX5PF015) | 981/985 (99%) |
| BE326_BF1_otu15 | *Silanimonas* sp. AK13 | [HE573746](http://www.ncbi.nlm.nih.gov/nucleotide/375340926?report=genbank&log$=nuclalign&blast_rank=11&RID=V5CCX5PF015) | 1305/1352 (97%) |
| BE326_BF1_otu16 | *Methylovulum miyakonense* HT12 | [AB501287](http://www.ncbi.nlm.nih.gov/nucleotide/295855440?report=genbank&log$=nuclalign&blast_rank=4&RID=V5CCX5PF015) | 1216/1340 (91%) |
| BE326_BF1_otu17 | Uncultured bacterium | [AF254393](http://www.ncbi.nlm.nih.gov/nucleotide/12584806?report=genbank&log$=nuclalign&blast_rank=1&RID=V5CCX5PF015) | 918/969 (95%) |
| BE326_BF1_otu18 | Uncultured bacterium | [FJ502258](http://www.ncbi.nlm.nih.gov/nucleotide/227016691?report=genbank&log$=nuclalign&blast_rank=1&RID=V5CCX5PF015) | 1317/1349 (98%) |
| BE326_BF1_otu19 | Uncultured bacterium | [HM748809](http://www.ncbi.nlm.nih.gov/nucleotide/302176482?report=genbank&log$=nuclalign&blast_rank=1&RID=V5CCX5PF015) | 1300/1348 (96%) |
| BE326_BF1_otu20 | Uncultured bacterium | [GQ354978](http://www.ncbi.nlm.nih.gov/nucleotide/256956176?report=genbank&log$=nuclalign&blast_rank=1&RID=V5CCX5PF015) | 1294/1301 (99%) |
| BE326_BF1_otu21 | Uncultured bacterium | [FJ517114](http://www.ncbi.nlm.nih.gov/nucleotide/219693591?report=genbank&log$=nuclalign&blast_rank=1&RID=V5F926SB01R) | 1342/1374 (98%) |
| BE326_BF1_otu22 | *Denitrovibrio acetiphilus* DSM 12809 | [CP001968](http://www.ncbi.nlm.nih.gov/nucleotide/290883107?report=genbank&log$=nuclalign&blast_rank=2&RID=V5F926SB01R) | 1288/1377 (94%) |
| BE326_BF1_otu23 | Uncultured bacterium | [AJ306780](http://www.ncbi.nlm.nih.gov/nucleotide/20269020?report=genbank&log$=nuclalign&blast_rank=1&RID=V5F926SB01R) | 1306/1361 (96%) |
| BE326_BF1_otu24 | Uncultured bacterium | [GU214148](http://www.ncbi.nlm.nih.gov/nucleotide/281313247?report=genbank&log$=nuclalign&blast_rank=1&RID=V5F926SB01R) | 1259/1310 (96%) |
| BE326_BF1_otu25 | Uncultured bacterium | [AB473887](http://www.ncbi.nlm.nih.gov/nucleotide/218774933?report=genbank&log$=nuclalign&blast_rank=1&RID=V5F926SB01R) | 1284/1306 (98%) |
| BE326_BF1_otu26 | Uncultured bacterium | [JN486832](http://www.ncbi.nlm.nih.gov/nucleotide/364681398?report=genbank&log$=nuclalign&blast_rank=1&RID=V5F926SB01R) | 1253/1300 (96%) |
| BE326_BF1_otu27 | Uncultured bacterium | [JN223427](http://www.ncbi.nlm.nih.gov/nucleotide/343175565?report=genbank&log$=nuclalign&blast_rank=1&RID=V5F926SB01R) | 1069/1167 (92%) |
| BE326_BF2_otu1 | *Thiobacillus thioparus* THI 115 | [HM535225](http://www.ncbi.nlm.nih.gov/nucleotide/302029724?report=genbank&log$=nuclalign&blast_rank=22&RID=V4R4T4PF01P) | 1331/1367 (97%) |
| BE326_BF2_otu2 | Uncultured bacterium | [GQ921470](http://www.ncbi.nlm.nih.gov/nucleotide/267798984?report=genbank&log$=nuclalign&blast_rank=1&RID=V4R4T4PF01P) | 1391/1396 (99%) |
| BE326_BF2_otu3 | *Thiofaba tepidiphila* BDA453 | [AB304258](http://www.ncbi.nlm.nih.gov/nucleotide/159576699?report=genbank&log$=nuclalign&blast_rank=7&RID=V4R4T4PF01P) | 1358/1362 (99%) |
| BE326_BF2_otu4 | *Thiovirga sulfuroxydans* SO07 | [AB118236](http://www.ncbi.nlm.nih.gov/nucleotide/44885723?report=genbank&log$=nuclalign&blast_rank=44&RID=V4R4T4PF01P) | 1351/1357 (99%) |
| BE326_BF2_otu5 | *Rhodobacter* sp. ZH15 | [FJ872532](http://www.ncbi.nlm.nih.gov/nucleotide/269819864?report=genbank&log$=nuclalign&blast_rank=4&RID=V4R4T4PF01P) | 1223/1281 (95%) |
| BE326_BF2_otu6 | *Rhodobacter* sp. TUT3732 | [AB251408](http://www.ncbi.nlm.nih.gov/nucleotide/111036419?report=genbank&log$=nuclalign&blast_rank=12&RID=V4R4T4PF01P) | 1274/1317 (97%) |
| BE326_BF2_otu7 | *Rhodobacter* sp. TUT3732 | [AB251408](http://www.ncbi.nlm.nih.gov/nucleotide/111036419?report=genbank&log$=nuclalign&blast_rank=12&RID=V4R4T4PF01P) | 840/889 (94%) |
| BE326_BF2_otu8 | *Stappia* sp. F2 | [JF899875](http://www.ncbi.nlm.nih.gov/nucleotide/335893105?report=genbank&log$=nuclalign&blast_rank=1&RID=V4R4T4PF01P) | 976/978 (99%) |
| BE326_BF2_otu9 | *Maricaulis maris* NBRC 102483 | [AB681822](http://www.ncbi.nlm.nih.gov/nucleotide/359805544?report=genbank&log$=nuclalign&blast_rank=18&RID=V4R4T4PF01P) | 1155/1297 (89%) |
| BE326_BF2_otu10 | Uncultured bacterium | [AM950258](http://www.ncbi.nlm.nih.gov/nucleotide/218117200?report=genbank&log$=nuclalign&blast_rank=1&RID=V4R4T4PF01P) | 947/955 (99%) |
| BE326_BF2_otu11 | Bacterium 4F6B | [AB623230](http://www.ncbi.nlm.nih.gov/nucleotide/328835492?report=genbank&log$=nucltop&blast_rank=13&RID=V4R4T4PF01P) | 1310/1350 (97%) |
| BE326_BF2_otu12 | Uncultured bacterium | [HQ755945](http://www.ncbi.nlm.nih.gov/nucleotide/319463084?report=genbank&log$=nuclalign&blast_rank=1&RID=V4R4T4PF01P) | 921/927 (99%) |
| BE326_BF2_otu13 | Uncultured bacterium | [JN825480](http://www.ncbi.nlm.nih.gov/nucleotide/365928030?report=genbank&log$=nuclalign&blast_rank=1&RID=V4R4T4PF01P) | 1243/1307 (95%) |
| BE326_BF2_otu14 | Uncultured bacterium | [AB473887](http://www.ncbi.nlm.nih.gov/nucleotide/218774933?report=genbank&log$=nuclalign&blast_rank=1&RID=V4R4T4PF01P) | 1272/1309 (97%) |
| BE326_BF2_otu15 | Uncultured bacterium | [GU214148](http://www.ncbi.nlm.nih.gov/nucleotide/281313247?report=genbank&log$=nuclalign&blast_rank=1&RID=V4R4T4PF01P) | 947/993 (95%) |
| BE326_BF2_otu16 | Uncultured bacterium | [GU214171](http://www.ncbi.nlm.nih.gov/nucleotide/281313270?report=genbank&log$=nuclalign&blast_rank=1&RID=V4R4T4PF01P) | 881/981 (90%) |
